# Prevalence and predictors of condom use among people who inject drugs in Georgia

**DOI:** 10.21203/rs.3.rs-4521575/v1

**Published:** 2024-06-27

**Authors:** Maia Kajaia, Maia Butsashvili, Jack A. DeHovitz, George Kamkamidze, Lasha Gulbiani, Tinatin Abzianidze, Mamuka DjibutiMD

**Affiliations:** Ivane Javakhishvili Tbilisi State University; Health Research Union (HRU); SUNY Downstate Health Sciences University; Health Research Union (HRU); Health Research Union (HRU); Health Research Union (HRU); Partnership Research Action Health (PRAH)

**Keywords:** condom, PWID, HIV, risky, behavior, consistent

## Abstract

**Background:**

People who inject drugs (PWID) are more likely to engage in risky sexual behavior placing them at high risk of acquiring HIV and other STIs. This study aims to assess the prevalence and predictors of inconsistent condom use with casual and/or paid sexual partners among PWID in Georgia.

**Methods:**

Integrated Bio-Behavioral Surveillance Survey was conducted among PWID in seven major cities of Georgia. Study design was cross-sectional with respondent-driven sampling (RDS) methodology. Data collection was carried out through individual face-to-face interviews. In this paper we analysed subsample of 619 PWID who reported having casual and/or paid sexual partners during last 12 months and described prevalence and predictors of consistent condom use.

**Results:**

Consistent condom use during casual and/or paid sex in past 12 months was reported by 49.4% of respondents. The likelihood of consistent use with casual and/or paid sexual partners was statistically significantly associated with residence, family income, drug use frequency, drug dependance and HIV risk self-perceptions. In multivariate analysis independent predictors of always using condom at casual/paid sex during the last 12 months were place of residence (aOR = 6.4; 95% CI: 3.2–12.7), family income (aOR = 2.1; 95% CI:1.3–3.5) and drug use frequency (aOR = 0.6; 95% CI: 0.4–0.9).

**Conclusion:**

The study revealed low prevalence of consistent condom use with casual and/or paid sexual partners among PWID in Georgia. Integration of safe sex educational interventions in harm reduction services will improve the rates of condom use among PWID and should focus PWID with lower socio-economic status and residing outside capital city.

## Introduction

Georgia is considered a low HIV prevalence country (estimated 0.4% HIV prevalence in the adult population) with cases of HIV infection concentrated among key affected populations including people who inject drugs (PWID) [1]. The country has one of the highest rates of injection drug use in the world [2]. National prevalence estimates for injection drug use are 2.23% in 18–64 years old population, and 1.39% in general population [3]. According to Georgian AIDS and Clinical Immunology Research Center 32.6% of HIV cases are among PWID [4].

Although parenteral exposure resulting from risky injection behavior is the main cause of HIV infection in people who inject drugs (PWID) [5, 6], a significant proportion of HIV infection in this population is sexually transmitted [7, 8]. PWID are more likely to engage in risky sexual behavior, placing them at high risk of acquiring HIV and other sexually transmitted infections (STIs)as well as subsequent transmission to non-drug-using sexual partners [9, 10, 11, 12, 13]. Correct and consistent use of condoms is an effective measure of preventing transmission of STIs including HIV [14, 15, 16]. While, condom use is one of the main interventions to prevent HIV infection for key affected populations and general public [17, 18, 19], interventions among PWID are mainly focused on reduction of injection risk behaviors [20, 21]. Unsafe injection practices can be significantly reduced with such interventions, but unsafe sexual behavior among PWID is difficult to modify [22, 23] and multiple studies show high rates of unprotected sex among PWID [24, 25, 26, 27, 28]. Given that PWID are at higher risk of HIV and other STIs compared to the general population they represent a vulnerable population who need specific approach addressing not only drug-related but also sexual risk behaviors. This study aims to assess the prevalence and predictors of inconsistent condom use with casual and/or paid sexual partners among PWID in Georgia.

## Methods

An Integrated Bio-Behavioral Surveillance Survey (IBSS) was conducted among PWID in seven major cities of Georgia: Tbilisi (capital city), Gori, Rustavi, Telavi, Batumi, Zugdidi, and Kutaisi. Study design was cross-sectional. Study participants were recruited by respondent-driven sampling (RDS) utilizing recruitment of research participants by other participants. The RDS method is based on social network theory and includes non-probability “snowball sampling” with mathematical modeling, which allows weighing the sample. This allows the investigation to get closer to representative estimates as much as possible [29]. The recruitment of study participants included a double incentive system: a primary reward for participating in the study and a secondary reward for recruiting other PWID into the study. The primary reward was 20 GEL (approximately 7 USD), and the secondary reward was 10 GEL (approximately 3.5 USD) for the inclusion of each new respondent in the study. Study participants were selected according to the following inclusion criteria: age ≥ 18 years, drug injection practice at least once in the 30 days prior to the survey, residence in the selected cities where the survey was conducted, willingness and ability to give informed consent for study participation. The assessment of potential study participants for being PWID was done by informal interview addressing drug prices, slang names, preparation, and injection techniques. In addition, objective signs of injecting drug use were assessed. Each eligible potential study participant was informed about the purpose, objectives, methods, procedures, risks, and benefits of the study. All individuals who agreed to participate in the study signed an informed consent form and then were enrolled in the study. The study included behavioral and biomarker components. The behavioral component data collection was carried out through individual, face-to-face interviews. The survey tool was a structured questionnaire collecting the following information: socio-demographic characteristics, injection practices, sexual behavior, use of HIV-preventive programs, and social factors related to drug use. The biomarker component of the study included testing of blood samples for HIV infection, hepatitis B and hepatitis C (the results of this component will be reported elsewhere).

Before initiation of field work, the study protocol and instruments were reviewed and approved by Institutional Review Board of Health Research Union (IRB00009520; IORG005619).

In total 2005 PWID participated in the study. In this paper we analysed a subsample of 619 PWID who reported having casual and/or paid sexual partners during the last 12 months and described prevalence and predictors of consistent condom use. “Casual sexual partner” was defined as a sexual partner who is not a regular partner and with whom a sexual relationship is established without financial compensation. “Paid sexual partner” was defined as a sexual partner with whom a sexual relationship is established in exchange for material remuneration (pays the partner or receives remuneration from the partner). “Consistent condom use” was defined as self-reported “always using condom” with casual and/or paid sexual partners during the last 12 months. Descriptive statistical methods were used to characterize socio-demographics, sexual behavior and condom use of study population. In bivariate analysis study variables were compared between different study groups (participants who consistently used condoms and those who did not) using chi-square test for categorized data. Logistic regression model was used for multivariate analysis to identify independent predictors of consistent condom use.

## Results

As noted above 619 PWID (30.7%) reported having casual and/or paid sex during the last 12 months. Almost all participants (n = 612, 98.9%) were males. The age of the respondents ranged from 18 to 67 years (median age 39 years). The vast majority of the study participants were ethnically Georgian (n = 605, 97.7%). 30.7% (n = 190) held a university degree. 33.6% (n = 208) of the surveyed PWID were married and almost two thirds (n = 384, 62.0%) were unemployed. Most of the respondents (n = 491, 79.4%) lived with their spouse/partner, parents, or relatives. Most of the study participants’ family income (n = 210, 55.9%) was less than 700 GEL (270 USD) per month (Table 1).

Table 2 describes sexual behavior and condom use with casual and paid sexual partners. Most of the respondents (n = 491, 79.9%) had their first sexual intercourse before the age of 18. The median age of sexual debut was 16 years. The majority of surveyed individuals (n = 396, 64.9%) had three or more sexual partners during the last 12 months. 329 (54.5%) of the study subjects reported using a condom during the last sexual intercourse with any sexual partner and 460 (74.3%) with casual and/or paid sex partner. Nearly two thirds of the interviewed PWID (n = 375, 61.8%) indicated that were under the influence of drugs during the last sexual contact. Most of the study subjects (n = 517, 83.5%) did not have any problems with obtaining condoms. Condom use during the last sexual intercourse was the result of a shared decision with the partner among 98 (46.4%) of PWID, in 68 (32.2%) - only the respondent’s decision, and in 29 (13.7%) - only the partner’s decision. The main reasons for not using a condom at last sexual contact were: “not considering necessary to use condoms” (n = 9, 42.9%) and “not liking to use condoms” (n = 5, 23.8%). Consistent condom use with casual and/or paid sex during the last 12 months was reported by only 306 (49.4%) of the respondents.

By bivariate analysis the likelihood of consistent condom use with casual and/or paid sexual partner was statistically significantly associated with residence, family income, drug use frequency, drug dependance and HIV risk self-perceptions. T probability of always using condom during casual/paid sex was higher among the respondents residing in Tbilisi (73.2%) compared to those living in other regional cities (43.3%) (OR = 3.5; CI: 2.3–5.5). PWID with family income ≥ 500 GEL (approximately 200 USD) were more likely to use condoms consistently with casual/paid sexual partners than those with lower income (57.3% vs 41.5%, OR = 1.8; 95% CI:1.2–2.9)). A lower proportion of the respondents who injected drugs frequently (once a week or more) always used condom at casual/paid sex compared to more rare injectors (once or several times per month) (46.7% vs 56.5%; OR = 0.6; 95% CI:0.4–0.9). PWID who didn’t perceive themselves as drug dependent (56.0%) were more likely to report consistent condom use at casual/paid sex, than those who did (45.8%) (OR = 1.5; 95% CI:1.1–2.1). HIV risk self-perception was also associated with consistent condom use, as higher percentage of the respondents who thought that they were under the risk of contracting HIV were always using condom with casual/paid sexual partners, compared to those who didn’t consider themselves at risk of HIV (50.8% vs. 35.7%; OR = 1.8; 95% CI: 1.1–3.2) (Table 3).

In multivariate analysis independent predictors of always using condom at casual/paid sex during the last 12 months were place of residence (aOR = 6.4; 95% CI: 3.2–12.7), family income (aOR = 2.1; 95% CI:1.3–3.5) and drug use frequency (aOR = 0.6; 95% CI: 0.4–0.9) (Table 3).

## Discussion

This study revealed high levels of risky sexual behavior among PWID in Georgia. The findings indicate that substantially higher proportion of the interviewed PWID had multiple sex partners (64.9% had ≥ 3 sexual partners) compared to other studies conducted in different countries where this indicator varied between 14–47% [30, 31, 32, 33, 34]. We also found a high prevalence of unprotected last sexual intercourse with any type of sexual partner among PWID and this finding is consistent with other studies [35, 36, 37, 38].

Consistent condom use during casual and/or paid sex was reported only by 49.4% of PWID. Other studies also showed the high rates of inconsistent condom use among PWID [38 39, 40, 41, 42]. Low rates of consistent condom use among PWID can be a consequence of sex under influence of drugs. Many PWID take drugs before sexual intercourse which can influence their decision and negotiation with partner on condom use. One important finding was that the frequency of drug use showed a positive association with inconsistent condom use. This finding is in line with previous studies suggesting that substance use leads to risky sexual behaviors [43, 44, 45 46]. Drug use might decrease the perception of risky behaviors and capacity to control these behaviors among PWID and thus could facilitate the engagement into risky sexual behaviors. It underlies the importance of enrollment and adherence to opioid substitution therapy (OST) among PWID, as being on OST, adhering to treatment and terminating injection drug use could lead to safer sexual practices and decreased risk of HIV and other STI transmission [47, 48].

We found that the study participants who had higher family income were more likely to use condoms constantly with casual and/or paid sexual partners. It seems that PWID with low family income cannot afford to buy condoms, thus it is very important among key populations to enhance programs that promote condom use not only through education but also increase access to condoms [49]. Our opinion is supported by a study conducted by Song YS et al finding that taking condoms from clinic stocks was the best predictor of condom possession, which in turn was the best predictor of condom use among men enrolled in drug treatment programs [50].

Our study also showed that living in other cities was associated with higher odds of inconsistent condom use compared to living in Tbilisi (capital city). IBSS survey conducted in 2009 among PWID in Georgia showed similar association with place of residence regarding inconsistent condom use and “dual risk behavior” defined as both unsafe injecting behavior at last injection and not using condom at last casual and/or paid sex [51, 52]. This means that PWID from regional areas of the country are still more likely to practice unsafe sexual behaviors suggesting the need for additional behavioral health education about safe sex practices among PWID residing outside the capital city. Health education targeted at individual’s risk self-perception, behavioral and normative beliefs would likely influence sexual risk behaviors among PWID. In addition, there must be an increased efforts to reduce drug use as previous studies have shown that behavioral health education in combination with OST have positive impact on reduction of HIV related risk behaviors [53, 54].

The study had some limitations. First, data were collected through face-to-face interviews, therefore may be subject to social desirability bias which is particularly problematic in studies involving sexual behavior, as respondents may not accurately answered some of the sensitive questions, either by underreporting stigmatized activities or by overreporting normative ones, if their actual behavior is considered socially unacceptable. Second, the findings rely on the study participants’ self-reported data which can be accompanied by recall bias, as the study participants may have had difficulties in recalling information about their sexual behavior in the past 12 months. Third, because of cross-sectional study design, it is not possible to make causal inferences.

## Conclusion

The study highlights low prevalence of consistent condom use with casual and/or paid sexual partners among PWID in Georgia. Integration of education about safe sexual practices into harm reduction services is an important component to decrease unsafe sexual practices and improve the rates of condom use among PWID in Georgia. Safe sex educational interventions should focus PWID with lower socio-economic status and residing outside capital city.

## Figures and Tables

**Table 1.** Socio-demographic characteristics

Characteristics	N	%
**Age**		
≤30 years	124	20
>30 years	495	80
Median age (min-max)	39 (18–67)	
**Gender**		
Male	612	98.9
Female	7	1.1
**Nationality**		
Georgian	605	97.7
Other	14	2.3
**Education**		
Primary (1– 4 classes)	5	0.8
Secondary (school, technical school, vocational school)	357	57.7
Incomplete university	64	10.3
University	190	30.7
Refused to answer	3	0.5
**Employment**		
Student	6	1.0
Have a permanent job	90	14.5
Have a temporary job	127	20.5
Retired/disabled	3	0.5
Unemployed	384	62.0
Refused to answer	9	1.5
**How much is your monthly income?**		
≤100 GEL	32	5.2
101–300 GEL	84	13.6
301–500 GEL	79	12.8
501–700 GEL	15	24.3
701–1000 GEL	68	11.0
>1000 GEL	143	23.1
Refused to answer	62	10.0
**What is your marital status?**		
Married	208	33.6
Divorced/Separated	161	26.0
Widow	6	1.0
Never been married	244	39.4
**With whom do you live now?**		
With spouse/partner	191	30.9
Alone	112	18.1
With parents/relatives	300	48.5
Refused to answer	16	2.6

**Table 2.** Sexual behavior and condom use

Characteristics	N	%
**How old were you when you had the first sexual intercourse?**		
<18 years old	491	79.9
≥18 years old	112	18.1
Don’t know	16	2.5
**Median age of beginning sexual life (min-max)**	16 (13–24)	
**In total, with how many sexual partners have you had during the last 12 months?**		
1	43	7.0
2	126	20.7
≥3	396	64.9
Don’t know/ No response	45	7.4
**Did you use condom during the last sexual intercourse?**		
Yes	329	54.5
No	263	43.5
Don’t know/No response	12	2.0
**Did you use condom during the last casual and/or paid sexual intercourse?**		
Yes	460	74.3
No	159	25.7
**Were you or your sexual partner under the influence of drugs during the last sexual intercourse?**		
Yes, I was	375	61.8
Yes, my sexual partner was	3	0.5
Yes, both me and my sexual partner were	32	5.3
No	153	25.2
Don’t know/No response	44	7.2
**Have you had any problem(s) obtaining condoms during the last month?**		
Yes	21	3.4
No	517	83.5
Don’t know	5	0.8
No response	76	12.3
**Whose decision was to use condom during the last sex?**		
My decision	68	32.2
Partner’s decision	29	13.7
Shared decision	98	46.4
Don’t know	16	7.6
**Why you didn’t use condom during the last sex?**		
The partner refused	1	4.8
Don’t like it	5	23.8
Don’t think it was necessary	9	42.9
Didn’t think of that	4	19.0
Other	1	4.8
**Frequency of condom use with casual and/or paid sex partner(s) in last 12 months**		
Always	306	49.4
Not always	313	50.6

**3.** Predictors of consistent condom use with casual and/or paid sexual partners

Characteristics	Consistent condom use with casual and/or paid sex partners		OR; 95% CI	*P value*	aOR; a95%CI	a*P value*
	N	%				
**Age**						
≤30 years	64	83.3				
>30 years	242	48.9	1.1 (0.7–16)	0.3		
**Marital status**						
Married	103	49.5	1.0 (0.7–14)	0.9		
Other	203	49.4				
**Level of education**						
High school/Vocational college	169	46.7	1.3 (0.9–1.8)	0.9		
University	136	53.5				
**Residence**						
Tbilisi	93	73.2	3.5 (2.3–5.5)	<0.0001	6.4 (3.2–12.7)	<0.0001
Other	213	43.3				
**Family Income**						
<500 GEL	81	41.5	1.8 (1.2–2.9)	0.02	2.1 (1.3–3.5)	0.002
≥500 GEL	86	57.3				
**Drug use frequency**						
Once or several times a month	121	56.5	0.6 (0.4–0.9)	0.02	0.6 (0.3–1.0)	0.07
Once a week or more	179	46.7				
**Alcohol consumption frequency**						
Never/Rarely	192	50.7	0.8 (0.6–1.2)	0.4		
Once a week or more	114	47.5				
**Drug dependance self-perception**						
Yes	202	45.8	1.5 (1.1–2.1)	0.02	1.5 (0.9–2.7)	0.9
No	89	56.0				
**HIV risk self-perception**						
Yes	280	50.8	1.8 (1.1–3.2)	0.03	2.2 (0.8–6.1)	0.1
No	20	35.7				
**Used preventive programs in last 1 year**						
Yes	168	56.4	1.3 (0.9–1.9)	0.9		
No	91	48.7				

## Data Availability

The data that support the findings of this study are not openly available due to reasons of sensitivity and are available from the corresponding author upon reasonable request. Data are located in controlled access data storage at Health Research Union, Tbilisi, Georgia.
